# Evaluating Sarcoid-Like Reactions in Melanoma Patients Treated With Pembrolizumab: A Systematic Review

**DOI:** 10.7759/cureus.103251

**Published:** 2026-02-09

**Authors:** Harras Khan, Fahd Mohamed, Jayyid Wafiy, Aditya Shah, Abimbola O Kolawole, Christopher Bobier

**Affiliations:** 1 College of Medicine, Central Michigan University, Mount Pleasant, USA; 2 Health Services Research, College of Medicine, Central Michigan University, Mount Pleasant, USA

**Keywords:** granulomatous inflammation, immune-checkpoint inhibitors, melanoma, pembrolizumab, prognostic biomarkers, sarcoid-like reactions

## Abstract

Pembrolizumab, a programmed cell death protein‑1 (PD‑1) inhibitor, has significantly improved outcomes in melanoma but is associated with immune‑related adverse events (irAEs), including sarcoid‑like reactions (SLRs). These granulomatous reactions can closely mimic metastatic disease on imaging, creating diagnostic uncertainty and potentially leading to unnecessary treatment changes.

This systematic review examined the incidence, clinical features, diagnostic strategies, management, and outcomes of SLRs in melanoma patients treated with pembrolizumab. A comprehensive search of PubMed, Scopus, and CINAHL identified 980 articles, of which 13 met eligibility criteria. Extracted data included patient demographics, SLR timing, organ involvement, radiographic patterns, histopathology, treatment approach, and clinical course.

The prevalence of SLRs ranged from 1.5% to 1.8%, with a mean onset of 7.1 months after therapy initiation. Pulmonary involvement, particularly hilar and mediastinal lymphadenopathy, was most frequently reported, followed by cutaneous and osseous granulomas. SLRs often mimicked melanoma progression on FDG‑PET/CT (fluorodeoxyglucose-positron emission tomography/computed tomography), emphasizing the importance of histologic confirmation. Most cases were managed conservatively or with corticosteroids, and pembrolizumab therapy was commonly continued without compromising melanoma control. Emerging evidence suggests that SLRs may reflect heightened immune activation and could potentially serve as favorable prognostic markers.

SLRs are uncommon but clinically important irAEs in pembrolizumab‑treated melanoma patients. Their ability to resemble metastatic disease underscores the need for diagnostic vigilance and routine tissue confirmation. Because SLRs are generally benign and may correlate with treatment response, standardized diagnostic and management protocols are needed to guide clinical decision‑making in this growing patient population.

## Introduction and background

Pembrolizumab, a monoclonal antibody targeting the programmed cell death protein (PD-1), has revolutionized cancer treatment by reactivating T-cell-mediated immune responses against tumor cells [1]. PD-1 is an inhibitory immune checkpoint receptor expressed primarily on activated T lymphocytes, as well as on B cells and natural killer cells, where it regulates peripheral immune tolerance. Pembrolizumab is a humanized monoclonal antibody that binds PD-1 and blocks its interaction with programmed death-ligand 1 (PD-L1) and PD-L2, thereby releasing inhibitory signaling and enhancing T-cell-mediated antitumor immune responses. Though initially approved for the treatment of advanced melanoma, its indications now extend to a broad range of malignancies, including non-small cell lung cancer, head and neck squamous cell carcinoma, and gastric cancer [1,2]. While the efficacy of pembrolizumab is well established, its mechanism of action can precipitate a range of immune-related adverse events (irAEs), among which sarcoid-like reactions (SLRs) in particular have gathered attention due to their clinical implications [3,4]. 

SLRs are characterized by non-caseating epithelioid cell granulomas and pose a diagnostic challenge due to their similarity to systemic sarcoidosis and their potential to mimic cancer progression [5]. Unlike true sarcoidosis, SLRs associated with pembrolizumab therapy usually lack systemic symptoms and tend to resolve upon discontinuation of the drug or with immunosuppressive therapy [2]. In reported cases, clinical or radiographic improvement typically begins within 2-6 weeks after discontinuation of pembrolizumab, with substantial regression over 2-3 months and near-complete resolution often observed by 3-6 months, particularly in the absence of ongoing immune stimulation or with adjunctive corticosteroid therapy. SLRs can span across multiple organ systems, including the lungs, lymph nodes, skin, and bones, and are typically asymptomatic or mildly symptomatic [3,6,7]. The pathophysiology behind pembrolizumab-induced SLRs is not well known but is proposed to be due to heightened T-cell activation, leading to the inadvertent targeting of healthy tissues. 

Despite being increasingly recognized, SLRs remain underreported in the literature, with most data derived from isolated case reports or small series [8]. Though limited, these reports highlight the need for increased awareness among clinicians to avoid misdiagnosing SLRs as disease progression, which could lead to unnecessary interventions or changes in cancer management. Moreover, there is limited guidance on the optimal management of SLRs, including whether to discontinue pembrolizumab therapy, initiate corticosteroids, or continue immunotherapy while monitoring the patient [2,4]. 

Given that pembrolizumab’s original indication was for advanced melanoma, this systematic review aims to consolidate the current body of evidence on pembrolizumab-induced SLRs and explore whether melanoma patients being treated with pembrolizumab raise a significant concern for developing SLRs. By synthesizing data from a range of case studies, this review seeks to provide a comprehensive resource for clinicians and researchers, addressing gaps in the literature and highlighting areas for future investigation. Furthermore, it underscores the importance of differentiating SLRs from true malignancy progression, especially given that pembrolizumab continues to be approved for a growing range of cancer types. 

## Review

Methods

Data Sources and Search Strategy

This study followed the Preferred Reporting Items for Systematic Reviews and Meta-Analyses (PRISMA) guidelines. A systematic review was conducted to identify all published reports describing sarcoid or SLRs associated with pembrolizumab therapy. A comprehensive electronic literature search was conducted in PubMed, Embase, Scopus, MEDLINE, and Google Scholar. Search terms included "pembrolizumab," "sarcoid-like reaction," "granulomatous reaction," "melanoma," and related terms.* *

Eligibility Criteria and Selection Process

**Figure 1:**
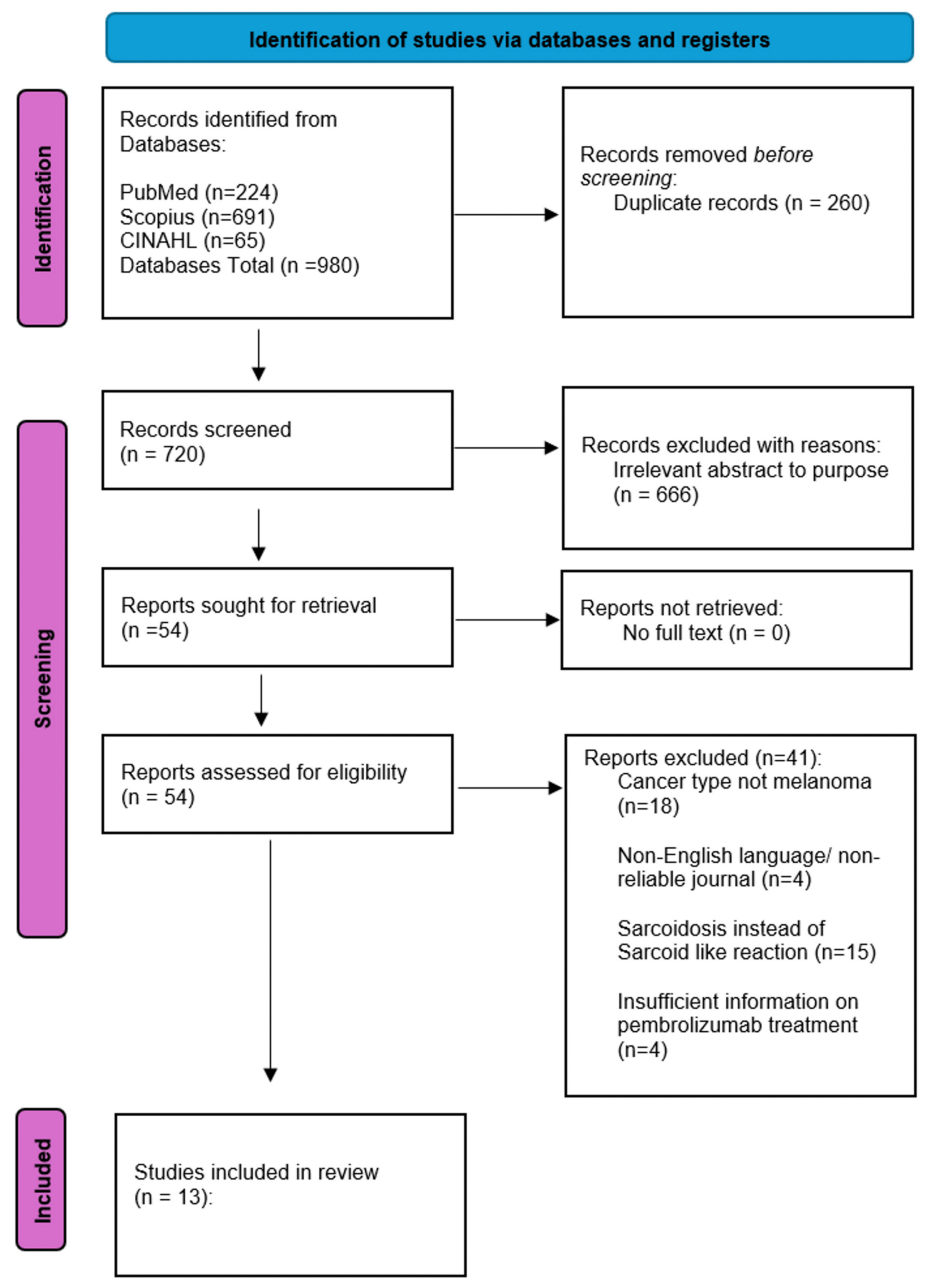
PRISMA Flowchart

Inclusion criteria were (1) human subjects, (2) a clear clinical description of sarcoid or sarcoid-like skin changes after pembrolizumab, (3) English language, and (4) case reports, case series, and observational studies. Exclusion criteria were (1) non-cutaneous adverse events, (2) reactions attributed to other immune checkpoint inhibitors (ICIs) without pembrolizumab involvement, and (3) insufficient clinical detail. Five independent reviewers screened titles and abstracts for relevance, followed by a full-text review. A PRISMA flow summary outlines the selection process (Figure 1).

Data Extraction

Data extraction was performed independently by two reviewers. Extracted variables included patient demographics, underlying malignancy, pembrolizumab dose and treatment duration, time from therapy initiation to onset of the sarcoid or SLR, clinical presentation, anatomic sites involved, imaging findings, histopathologic characteristics, management strategies (continuation or discontinuation of pembrolizumab, corticosteroid use, additional therapies), and clinical outcomes.* *Discrepancies between reviewers were resolved through discussion and consensus. 

Risk of Bias/Quality Assessment

A structured risk-of-bias assessment was performed for all included studies in accordance with PRISMA 2020 recommendations. Because the evidence base consisted primarily of single-patient case reports and small case series, study quality was evaluated using the Joanna Briggs Institute (JBI) Critical Appraisal Checklists for Case Reports and Case Series, respectively. These tools assess clarity and completeness across domains, including patient history, clinical presentation, diagnostic workup, intervention details, follow-up, and clinical reasoning. Each study was independently appraised by two reviewers, with discrepancies resolved by consensus. For the pharmacovigilance-based database study and the previously published systematic review identified during screening, quality appraisal was performed using an adapted JBI analytical cross-sectional checklist and AMSTAR-2, respectively; however, these studies were not included as primary evidence because they did not meet the predefined inclusion criteria requiring patient-level pembrolizumab-associated SLRs. Given that case reports and case series inherently carry a high risk of publication and selection bias, risk-of-bias determinations were used to inform qualitative interpretation rather than to exclude studies. No studies were removed on the basis of quality alone.

Data Synthesis

Due to the limited number of cases and heterogeneity of reporting, a narrative, qualitative synthesis was undertaken. Findings were organized thematically according to incidence, clinical characteristics, diagnostic approaches, management strategies, and outcomes. Findings were organized thematically according to incidence, clinical characteristics, diagnostic approaches, management strategies, and outcomes. When possible, descriptive statistics (e.g., medians, ranges, and simple proportions) were used to summarize trends such as time to onset, organ involvement patterns, and treatment responses. Heterogeneity in study design, diagnostic thresholds, and reporting practices was explicitly considered when interpreting patterns across cases.

Results 

Study Selection

Our search identified a total of 980 papers. After removing duplicates, 254 articles were excluded at the title screening stage, and 412 were excluded based on abstract review. This resulted in 54 full-text articles for detailed evaluation. Studies were excluded if they focused on non-melanoma cancer types (18 articles), were non-English or published in non-reliable journals (four articles), discussed sarcoidosis without relevance to SLRs (15 articles), or lacked sufficient information on pembrolizumab treatment (four articles). Ultimately, 13 articles were included in this systematic review, comprising nine case reports, one case series, two clinical trials, and two database syntheses (Figure 1). Of the 13 studies included in this review, the majority were single-patient case reports (n=9, 69%), with one case series and three contextual studies. Using the JBI Critical Appraisal tools, most case reports demonstrated a low risk of bias, characterized by clear clinical timelines, adequate diagnostic evaluation, and consistent histopathologic confirmation of sarcoid-like granulomatous inflammation. The single case series was rated as moderate risk of bias, primarily due to small sample size and limited control of confounders.

Narrative Synthesis

Across the included articles, four recurring themes were identified regarding SLRs in melanoma patients receiving pembrolizumab: incidence and characteristics, diagnostic approaches, management and outcomes, and prognostic implications. Three articles addressed all four themes, while the remaining 10 addressed at least two. Table 1 highlights the characteristics of the studies.

**Table 1:** Characteristics of Included Studies on Pembrolizumab-Associated Sarcoid-Like Reactions Key characteristics of the included studies, including study design, population, pembrolizumab exposure, organ involvement, diagnostic approach, management, and outcomes. Studies marked with an asterisk (*) were used as contextual background and were not part of the primary-level evidence synthesis. FDG-PET/CT, fluorodeoxyglucose-positron emission tomography/computed tomography; ICIs, immune checkpoint inhibitors; EBUS, endobronchial ultrasound; SLR, sarcoid-like reactions; irAEs, immune-related adverse events; ILD, Interstitial lung disease; PD-1, programmed cell death protein-1.

First Author (Year)	Country	Study Design	Sample Size	Cancer Type	Intervention/Exposure	SLR Site(s)	Diagnostic Method	Management	Key Outcomes
Gary (2022) [2]	USA	Case report (conference abstract)	1	Lung adenocarcinoma and melanoma	Pembrolizumab + chemotherapy	Lung; mediastinal lymph nodes	EBUS + CT-guided biopsy (granulomas; stains negative)	Steroids; immunotherapy held	Regression of granulomatous lesions on follow-up imaging
Woodbeck (2018) [3]	USA	Case report	1	Metastatic melanoma	Pembrolizumab	Cutaneous lesion (granulomatous tumoral melanosis)	Dermatopathology (granulomas; no melanoma)	Continued therapy with surveillance	Lesion represented granulomatous regression rather than progression
Chan (2023) [4]	USA	Case report (conference abstract)	1	Localized melanoma	Pembrolizumab (adjuvant)	Hilar lymph nodes; pulmonary nodules	Bronchoscopy + biopsy (non-necrotizing granulomas)	Pembrolizumab discontinued	Diagnosis prevented misclassification as metastasis
McKenna (2018) [6]	USA	Case report	1	Stage IIIC melanoma	Pembrolizumab	Cutaneous scars; lung; lymph nodes	Biopsy of FDG-avid lesion; EBUS negative for malignancy	Conservative/observation	Avoided overstaging; stable disease reported
Rubio-Rivas (2020)* [7]	Spain	Systematic review (contextual)	91 patients	Melanoma	ICIs ± BRAF/MEK inhibitors (includes pembrolizumab)	Multiorgan	Histology (as reported in included cases)	Variable	Descriptive synthesis; SLRs frequently compatible with disease control
Delaunay (2017)* [9]	France	Observational cohort (contextual)	—	Mixed cancers	Immune checkpoint inhibitors	Pulmonary irAEs (ILD; contextual)	Imaging ± biopsy (as reported)	Variable	Contextual pulmonary toxicity data; not patient-level pembrolizumab-SLR evidence
Chahin (2020) [10]	USA	Case report	1	Melanoma	Pembrolizumab	Multistation lymphadenopathy	EBUS biopsy (non-caseating granulomas; no malignancy)	Steroids; pembrolizumab resumed	Near-complete resolution of lymphadenopathy
Rodriguez (2019) [11]	USA	Case series	5	Melanoma	ICIs (includes 1 pembrolizumab + CMP-001)	Mediastinal and/or periportal lymph nodes	EBUS/EUS-FNA (granulomatous inflammation; stains negative)	Steroids ± ICI discontinuation	Resolution or marked improvement of lymphadenopathy in all cases
Izzedine (2022)* [12]	France	Narrative/database synthesis (contextual)	4,265 reports	Mixed cancers	ICIs including pembrolizumab	Multiorgan	Pharmacovigilance coding; disproportionality analysis	Variable	Signal supports association; incidence not estimable
Henderson (2022) [13]	USA	Case report (conference abstract)	1	Nasal mucosal melanoma	Pembrolizumab (adjuvant)	Lung; lymph nodes; bone	Biopsy of hip lesion (non-caseating granulomas; stains negative)	Corticosteroids	Radiographic regression of lesions reported
Jespersen (2018) [14]	Denmark	Case report	1	Metastatic melanoma	Pembrolizumab	Mediastinal lymph nodes; bone	FDG-PET/CT + biopsy	Corticosteroids	Resolution of SLR; complete oncologic response
Al-Dliw (2017) [15]	UK	Case report	1	Melanoma	Pembrolizumab	Pulmonary parenchyma	Transbronchial biopsy; infectious workup negative	High-dose corticosteroids	Rapid clinical and radiographic improvement
De Keukeleire (2020) [16]	Belgium	Case report	1	Metastatic melanoma	Pembrolizumab (anti–PD-1)	Mediastinal lymph nodes; liver hilum; bone	FDG-PET/CT + biopsy (non-caseating granulomas)	Observation; pembrolizumab discontinued	Spontaneous resolution of SLR; sustained melanoma remission

Table 2 highlights the quality assessment of the studies.

**Table 2:** Quality Appraisal Summary of Included Studies (n = 13) JBI, Joanna Briggs Institute; SLR, sarcoid-like reactions.

Study Design	Number of Studies	Appraisal Tool Used	Overall Quality Summary
Case reports	9	JBI Case Report Checklist	Majority rated low risk of bias with clear reporting of clinical course, diagnostic workup, and management; limitations primarily due to inherent single-patient design and incomplete long-term follow-up.
Case series	1	JBI Case Series Checklist	Moderate risk of bias due to small sample size and limited control of confounding; diagnostic procedures consistently reported.
Clinical trials reporting SLRs	2	Not appraised due to lack of patient-level SLR data	Not included in quality grading; used only for contextual information.
Pharmacovigilance/Database study	1	Adapted JBI Analytical Cross-Sectional Checklist	Moderate-high risk of bias for causal inference; used for contextual epidemiologic signal only.

Incidence and characteristics: The estimated prevalence of SLRs among melanoma patients treated with pembrolizumab ranged from 1.5% to 1.8%, with symptom onset occurring within weeks to months after treatment initiation (mean: 7.1 months) [7,9]. SLRs were characterized by non-caseating granulomatous inflammation, most commonly involving the lungs and mediastinal lymph nodes, although cutaneous and skeletal involvement was also documented [9].

Pulmonary manifestations were the most frequently reported. Imaging often demonstrated hilar and mediastinal lymphadenopathy or hypermetabolic lesions on FDG-PET/CT (fluorodeoxyglucose-positron emission tomography/computed tomography), frequently mimicking metastatic melanoma. For example, Chahin et al. described a 61-year-old male with stage IIB melanoma who developed widespread lymphadenopathy after six pembrolizumab cycles; biopsy revealed non-caseating granulomas, confirming an SLR rather than metastasis [10]. A similar presentation was reported in a 66-year-old female with diffuse pulmonary nodules and hilar adenopathy, in whom biopsy again confirmed pembrolizumab-associated granulomatous inflammation [2].

Diagnostic approaches: Cutaneous SLRs contributed to diagnostic complexity. McKenna et al. reported granulomatous inflammation localized to decade-old scars in a 69-year-old female shortly after treatment initiation [6]. Variability in SLR presentation, affecting skin, lung, lymph nodes, and bone, has been emphasized across multiple studies, necessitating a high index of suspicion in immunotherapy-treated patients [1,11,12]. Unique presentations such as granulomatous tumoral melanosis, characterized by pigment-laden histiocytes mimicking melanoma progression, reinforced the need for histopathological confirmation before modifying therapy [3]. Across all included studies, biopsy remained the diagnostic gold standard, consistently demonstrating non-caseating granulomas without evidence of malignancy or infection [13]. Ancillary tests, including serum angiotensin-converting enzyme (SACE) levels, were inconsistently elevated and deemed unreliable as diagnostic markers [6]. FDG-PET/CT commonly showed hypermetabolic lesions resembling metastases, highlighting the necessity of tissue sampling for definitive diagnosis [1,12,14].

Management and outcomes: SLR management varied by severity. Mild reactions were often monitored without discontinuing pembrolizumab, with many cases remaining stable or resolving spontaneously. Moderate to severe reactions, particularly those with significant pulmonary involvement, were typically treated with systemic corticosteroids, which produced rapid clinical and radiographic improvement. For instance, a 65-year-old female with worsening hypoxia and pulmonary infiltrates experienced marked improvement after corticosteroid initiation [15], similar to the patient reported by Gary and Egan [2]. In some cases, SLRs resolved following pembrolizumab discontinuation alone, supporting their occasionally self-limiting nature [4]. Importantly, Jespersen et al. [14] reported that appropriate management, whether through corticosteroids or therapy modification, did not compromise melanoma control, supporting the overall benign course of SLRs when recognized early and treated appropriately.

Prognostic implications: Emerging evidence suggests that SLRs may serve as a favorable prognostic marker, reflecting heightened immune activation. Studies have hypothesized that granulomatous reactions may correlate with an effective antitumor response [13]. De Keukeleire et al. described cases of sustained melanoma remission following SLR development, further supporting the possibility of favorable prognostic implications [16].

Discussion

This systematic review examined the incidence, clinical characteristics, diagnostic strategies, management approaches, and prognostic significance of SLRs in melanoma patients treated with pembrolizumab. Across the included studies, four major themes consistently emerged: (a) SLRs are uncommon but clinically important; (b) distinguishing SLRs from metastatic progression remains a major diagnostic challenge; (c) management is generally effective and does not appear to compromise melanoma control; and (d) SLRs may carry prognostic information related to immune activation. These findings support the deliberate recognition of SLRs as a distinct clinical entity within ICI therapy.

Comparison With Prior Evidence

Our findings are broadly consistent with prior evidence. As reported by Henderson et al. [13], SLRs most commonly present as non-caseating granulomatous inflammation involving the lungs and mediastinal lymph nodes, often closely resembling metastatic disease on CT or FDG-PET/CT. Multiple reports in our review demonstrate that radiographic findings alone cannot reliably distinguish SLRs from melanoma progression, underscoring the necessity of biopsy for accurate diagnosis. Management patterns also align with prior studies, including those by Gary and Egan [2], in which corticosteroids effectively resolved symptoms without apparent compromise of melanoma control. Furthermore, emerging data from De Keukeleire et al. [16] and others support the hypothesis that SLRs may reflect heightened antitumor immune activation and may correlate with sustained disease control.

Explaining Heterogeneity

Considerable heterogeneity among included studies limited comparability and synthesis. Variations in study design (e.g., case reports vs. clinical trial data), lack of standardized SLR definitions, differing diagnostic thresholds, and inconsistent imaging protocols all contributed to variability. Biopsy indications and follow-up durations were similarly inconsistent across reports. Patient-level factors, such as prior systemic therapies, baseline immune status, comorbidities, and tumor burden, likely further influenced presentation and outcomes. Reporting bias may also play a role, as unusual or severe cases are more likely to be published, potentially exaggerating the perceived diversity of SLR manifestations. These sources of heterogeneity reinforce the need for standardized diagnostic criteria and reporting frameworks in future studies.

Clinical Implications

Clinicians should maintain vigilance for SLRs throughout pembrolizumab therapy, particularly around the typical onset of approximately seven months. Given the frequent radiographic overlap with metastatic disease, histologic confirmation remains essential when imaging findings would alter management. Mild, asymptomatic SLRs can often be monitored while continuing pembrolizumab, whereas systemic corticosteroids should be reserved for symptomatic or organ-threatening involvement, consistent with the management approaches described by Gary and Egan [2]. Finally, growing evidence, including observations by De Keukeleire et al. [16], suggests that SLRs may represent a marker of robust immune activation. Recognizing this possibility may help clinicians balance oncologic benefit against the risks of treatment interruption or immunosuppressive therapy.

Quality of Evidence and Interpretation of Findings

Interpretation of the findings from this review must account for the overall quality of the available evidence. The majority of included studies were single-patient case reports with inherently limited generalizability, potential selection bias, and incomplete long-term follow-up. Nevertheless, these reports consistently demonstrated high internal validity, characterized by clear clinical timelines, robust diagnostic evaluations including histopathology, and detailed documentation of treatment courses. The sole case series offered additional insight into nodal SLRs but remained constrained by sample size and heterogeneity in prior therapies. Larger observational studies and pharmacovigilance analyses provided supportive contextual data but lacked the patient-level detail required for inclusion as primary evidence. Taken together, the evidence base supports the recognition of pembrolizumab-associated SLRs as a reproducible clinical phenomenon, while emphasizing the need for cautious interpretation given the predominance of low-level evidence.

Strengths and limitations

This review has several notable strengths. A comprehensive, multi-database search strategy was employed, supplemented by manual reference screening to maximize study capture. Predefined eligibility criteria and a structured, dual independent review process for study selection and data extraction helped minimize selection and extraction bias. Additionally, the synthesis incorporated clinical, radiologic, and pathologic dimensions of SLRs, allowing for a more integrated understanding of these reactions across diverse presentations. However, this systematic review is limited by the quality and nature of the available evidence. Most included studies were isolated case reports, which carry inherent risks of reporting bias, selective publication, and limited generalizability. Variability in diagnostic rigor, including differences in biopsy practices, imaging thresholds, and follow-up duration, introduced further heterogeneity across studies. Few reports provided uniform laboratory data or standardized criteria for defining SLRs, restricting comparability. In the absence of controlled studies, causal attribution between pembrolizumab exposure and granulomatous inflammation remains probabilistic rather than definitive. Despite these limitations, the consistent histopathologic confirmation and reproducible clinical patterns across studies strengthen the overall conclusions and underscore the clinical relevance of these reactions.

Recommendations for future research

Future studies should focus on prospective, multicenter cohorts and registries that use standardized case definitions, imaging criteria, and biopsy indications to reduce diagnostic variability in SLRs. Development of non-invasive diagnostic tools, including circulating immune biomarkers, cytokine panels, and more specific PET tracers, is needed to improve differentiation between SLRs and melanoma progression.

Mechanistic research using advanced immunophenotyping, T-cell receptor analyses, and host genetic studies could clarify why only some patients develop SLRs and whether these reactions correlate with stronger antitumor responses. Additionally, prospective evaluations of standardized management strategies, including the role and optimal use of corticosteroids, are necessary to understand their impact on melanoma outcomes.

Finally, longitudinal studies and pragmatic trials should assess the natural history of SLRs, recurrence risk, and long-term effects, providing evidence-based pathways that support accurate diagnosis while preserving oncologic efficacy.

## Conclusions

SLRs are uncommon but clinically important irAEs in melanoma patients receiving pembrolizumab. Their close radiographic resemblance to metastatic disease, particularly on FDG-PET/CT, and their diverse organ involvement create significant diagnostic uncertainty. In this setting, histologic confirmation remains essential, with non-caseating granulomas providing the most reliable diagnostic hallmark. Most SLRs respond well to conservative management or short courses of corticosteroids, and current evidence indicates that treatment of SLRs does not compromise melanoma control. Emerging data further suggest that SLRs may reflect heightened immune activation and could represent markers of a favorable antitumor response rather than simple toxicity.

Nonetheless, major gaps persist regarding the biological mechanisms driving SLR development and the absence of reliable non-invasive diagnostic tools. Prospective studies are needed to clarify the prognostic significance of SLRs, identify biomarkers capable of distinguishing SLRs from true progression, and establish standardized monitoring and management pathways. Ultimately, timely recognition and appropriate management of SLRs allow patients to continue benefiting from pembrolizumab without unnecessary treatment alterations. As understanding advances, SLRs may increasingly be viewed not only as diagnostic challenges but also as potential indicators of therapeutic efficacy in melanoma care.
